# Perceptions and attitudes toward participation in clinical research in the Eastern Mediterranean Region: A systematic review

**DOI:** 10.1097/MD.0000000000029959

**Published:** 2022-08-19

**Authors:** Mohamed Aabdien, Ibtihal Abdallah, Mohamed Iheb Bougmiza, Timo Siepmann, Ben Illigens

**Affiliations:** a Community Medicine Training Program: Medical Education, Hamad Medical Corporation, Doha, Qatar; b Division of Health Care Sciences, Center for Clinical Research and Management Education Dresden International University, Dresden, Germany; c Department of Pharmacy, Hamad Medical Corporation, Doha, Qatar; d Workforce Development & Training – Family & Community Medicine Department, Primary Health Care Corporation, Doha, Qatar; e Department of Neurology, University Hospital Carl Gustav Carus, Technische Universität Dresden, Dresden, Germany; f Department of Neurology, Beth Israel Deaconess Medical Center, Harvard Medical School, Boston, MA.

**Keywords:** attitude, clinical research, EMRO, involvement, participation, perception

## Abstract

**Introduction:**

Successful recruitment of participants into clinical research has always been challenging and is affected by many factors. This systematic review aimed to explore the perceptions and attitudes as well as identify the factors affecting the participation in clinical research among the Eastern Mediterranean Regional Office countries’ population.

**Methods:**

A systematic search of the literature was conducted to explore attitudes or perceptions of the general public or patients towards participation in clinical research. PubMed, Pro-Quest Central, World Health Organizations Index Medicus for the Eastern Mediterranean Region, and Google Scholar were searched. Studies were considered eligible for inclusion if they presented primary data and were conducted in one of the Eastern Mediterranean Regional Office countries. A data extraction sheet was used to record the following: year, country, aim, population, sample size, study design, data collection, and setting. The identified factors from the included studies were categorized into motivators and barriers.

**Results:**

In total, 23 original research articles were identified that addressed perceptions or attitudes towards clinical research participation. Six main motivators and barriers of research participation among patients, the general public, and patient family members were identified. The most common cited motivators included personal benefits to the individual, altruism and the desire to help others, the research process, the influence of the physician, family encouragement, and religion. Concerns regarding safety, confidentiality, and other factors in addition to the research process, lack of trust in healthcare providers or healthcare system, lack of interest in research and no perceived personal benefit, religious concerns, and family/cultural concerns were the most cited barriers to participation.

**Conclusion:**

The identified motivators and barriers are essential to tackle during clinical research planning among the population of Eastern Mediterranean Regional Office countries. Further research is needed to assess the attitudes and perceptions of individuals approached to participate in trials.

## 1. Introduction

Evidence-based medicine (EBM) is the practice that advocates the thorough examination of medical literature to extract the best available evidence when making clinical decisions. Such evidence is made available through the different types of research, including interventional and observational studies.^[1,2]^

Successful recruitment and retention of participants into clinical research have always presented as a challenge since the general public and patients might lack the awareness about the importance of clinical research for the advancement in healthcare and many other influencing factors.^[3]^ This might result in failing to meet recruitment targets, and sometimes, failure or termination of trials. The literature shows that >50% of oncology trials were terminated prematurely due to a low recruitment rate.^[4,5]^

The World Health Organization (WHO) Eastern Mediterranean Region Office (EMRO) comprises a diverse population from 22 countries. This population shares numerous similar demographics, religious, and cultural characteristics, yet diverse socioeconomic, racial, and ethnic backgrounds. Additionally, this region is changing population size and health-related characteristics. Hence, the need for expanding clinical research is crucial to face the challenges relating to healthcare provision. However, participation in clinical research in the EMRO countries is yet underdeveloped. A study by Nair et al showed that the participants from the region countries accounts for <1% of the global research participants’ size and 0.5% of the total global sites of clinical trial.^[6,7]^

In order to improve participation in clinical research, there is a need to explore the perceptions and attitudes of the general public to tackle their concerns when intending to recruit them to clinical research. Hence, this systematic review aimed to explore the perceptions and attitudes and identify the factors affecting the participation in clinical research among the EMRO countries population.

## 2. Methods

### 2.1. Search strategy

This review is registered on PROSPERO International Prospective Register of Systematic Reviews (registration number CRD42020195763). A comprehensive systematic literature search was conducted and reported following the Preferred Reporting Items for Systematic Reviews and Meta-Analyses (PRISMA) guidelines.^[8]^ The following databases were searched: PubMed, Pro-Quest Central, World Health Organizations Index Medicus for the Eastern Mediterranean Region (IMEMR), and Google Scholar. The databases were searched up to October 2021 using the following MeSH terms and keywords that were agreed upon by the authors: “research” AND “attitude” AND “involvement” OR “participation” AND the individual countries of the Eastern Mediterranean Region, a total of 22 countries (Afghanistan, Bahrain, Djibouti, Egypt, Iran, Iraq, Jordan, Kuwait, Lebanon, Libya, Morocco, Oman, Pakistan, Palestine, Qatar, Saudi Arabia, Somalia, Sudan, Syria, Tunisia, United Arab Emirates, and Yemen). As the search terms were broad, the search yielded many studies. Hence, we limited it to studies conducted in humans and published in English. Additionally, a manual search of the references list of the identified relevant articles was done to supplement the search.

### 2.2. Selection criteria

#### 2.2.1. Inclusion criteria.

Studies were included if they met the following criteria: presented original and primary research; explored attitudes or perceptions towards participation in clinical research among patients or the general public; conducted in one of the 22 countries of the EMRO region; and using a recognized method for data collections, such as questionnaire and structured interviews.

#### 2.2.2. Exclusion criteria.

Studies were excluded if they: did not meet all inclusion criteria or did not present original/primary research, such as reviews.

### 2.3. Data extraction and quality assessment

**Figure 1.**
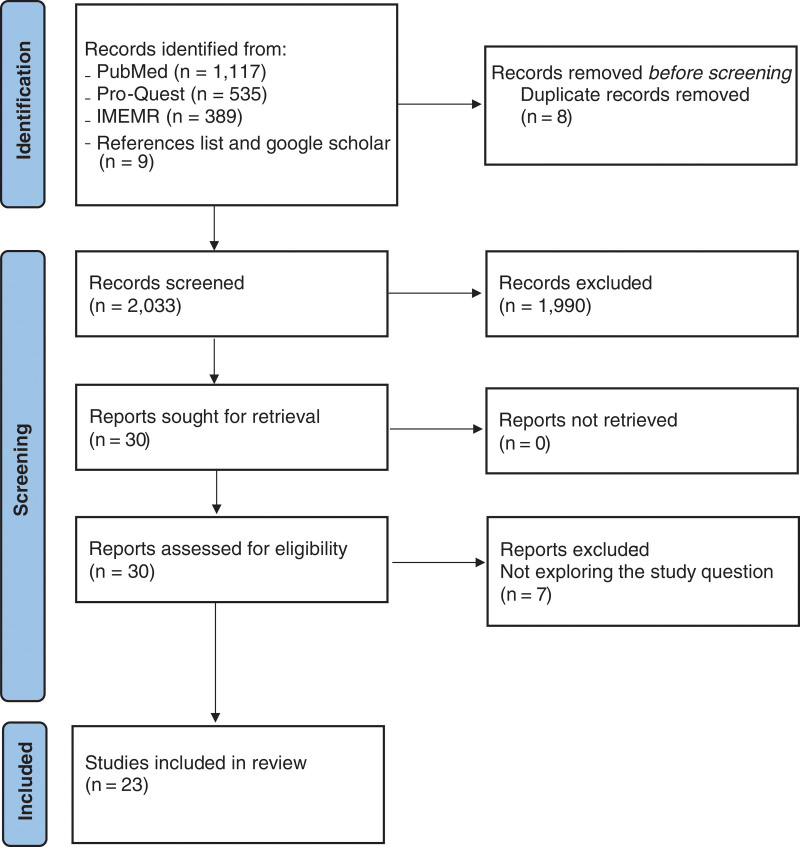
Flowchart of the study selection process for the systematic review following the Preferred Reporting Items for Systematic Reviews and Meta-Analyses guidelines (PRISMA).^[8]^

The titles of the articles retrieved by the initial search (Fig. 1) were independently screened by 2 authors. Those deemed relevant were further examined by reviewing the abstracts. After that, relevant abstracts were selected to be screened in full text. The full-text appraisal was independently performed by 2 authors using the following: The Newcastle-Ottawa Scale (NOS) for cross-sectional studies, adapted from the scale for cohort studies,^[9]^ and Critical Appraisal Skills Programme (CASP) for qualitative studies,^[10]^ aiming to ensure the quality of the included studies. In the case of disagreement, it was resolved through discussion among the authors. A coding template was developed to extract the data and categorize it into motivators and barriers to research participation among patients and the general public in the countries of the EMRO region.

## 3. Results

The systematic literature search across the databases yielded a total of 2041 studies, in addition to 9 articles that were identified through the supplemental search of the bibliographies of relevant articles (Fig. 1). Duplicates were subsequently removed, and 2033 studies were screened by title. The title screen yielded 44 studies that were to be assessed by abstract. Fourteen abstracts were excluded as they did not address the study question or were conducted among a population other than the targeted population. Thirty studies were assessed independently as full-text articles for meeting the inclusion criteria. At this stage, 7 articles were excluded for not exploring the perception/attitude toward participation and enrolling in research in the EMRO countries (n = 7). Twenty-three articles were agreed upon by the authors to be included in the review as they addressed the study question and met all the inclusion criteria. Table 1 presents all studies’ demographic and methodological characteristics included in the review.

**Table 1:** Studies included in the systematic review.

Study	Year	Country	Aim	Population	Sample size	Study design	Data collection	Setting
Abou-Zeid et al^[11]^	2010	Egypt	To determine the attitudes of patients regarding their participation in research and with the collection, storage and future use of blood samples for research purposes.	Adult patients	600	Cross-sectional survey	Interview questionnaire	Outpatient clinics
Ahram et al^[12]^	2014	Jordan	To assess factors influencing public participation in biobanking	Adult population	3196	Nationwide, cross-sectional survey	Interview survey	Household
Ahram et al^[13]^	2020	Jordan	To assess public knowledge of and willingness to participate in clinical trials and the associated sociodemographic characteristics	Adult population	3196	Population-based survey	Interview questionnaire	Household
Al-Amad et al^[14]^	2014	Jordan	To explore the attitudes of individuals regarding their decision to participate in dental research and the associated socioeconomic factors	Adult patients	120	Cross-sectional survey	Self-administered questionnaire	Dental clinics
Al-Dakhil et al^[15]^	2016	Saudi Arabia	To evaluate and investigate the beliefs and attitudes of patients in Saudi Arabia regarding participation and enrollment in the clinical trials	Adult patients	1081	Cross-sectional survey	Self-administered questionnaire	Hospital
Al-Jumah et al^[16]^	2011	Saudi Arabia	To determine the attitude to research involving storage and use of human tissues in genetic research and biobanks	Adult patients	1051	Cross-sectional survey	Interview questionnaire	Outpatient clinics
Al-Lawati et al^[17]^	2018	Oman	To assess the knowledge and attitudes of patients and their relatives towards participating in clinical trials	Adult patients and relatives	100	Cross-sectional survey	Self-administered questionnaire	Hospital and outpatient clinics
AL-Tannir et al^[18]^	2016	Saudi Arabia	To assess the knowledge, attitudes, and perceptions towards participating in clinical trials	Patients and companions	232	Cross-sectional survey	Self-administered questionnaire	Outpatient clinics
Bazarbashi et al^[19]^	2015	Saudi Arabia	To explore the perception, knowledge, and willingness of cancer patients to participate in oncology clinical trials	Patients and family	204	Cross-sectional survey	Interview questionnaire	Outpatient clinics
El Obaid et al^[20]^	2016	United Arab Emirates	To explore participants general perceptions towards medical research	Adult population	42	Qualitative study design	Focus groups	Academic institutions & blood bank
Gharaibeh et al^[21]^	2020	Jordan	To assess the willingness of patients to participate in clinical trials, and patients’ attitudes, perceptions to research	Adult patients	1201	Cross-sectional survey	Interview questionnaire	Hospital
Hassona et al^[22]^	2016	Jordan	To study the willingness of dental patients to donate biospecimens for research purpose and to examine decisions influencing factors	Adult patients	408	Cross-sectional survey	Interview questionnaire	Hospital
Hifnawy et al^[23]^	2017	Egypt, Lebanon, Saudi Arabia & Sudan	To determine patients’ attitudes and perceptions toward research participation and perceptions of their rights	Adult patients	202	Cross-sectional survey	Interview questionnaire	Outpatient clinics & hospital
Khalil et al^[24]^	2007	Egypt	To examine the attitudes of Egyptian individuals toward medical research	Patients and family	15	Qualitative study design	Semistructured interview	Outpatient clinics
Killawi et al^[25]^	2014	Qatar	To describe procedures related to recruiting, obtaining informed consent, and compensating health research participants	Adult patients	84	Qualitative study design	Field observations & interviews	Outpatient clinics
Lhousni et al^[26]^	2020	Morocco	To explore knowledge and attitude toward biobanks among patients and to evaluate their willingness to donate their own biological samples	Adult patients	1133	Cross-sectional survey	Interview questionnaire	Health care centers
Makhlouf et al^[27]^	2019	Jordan	To explore and understand population’s perspectives, expectations, and concerns toward biobanks	Adult Population	500	Cross-sectional survey	Self-administered questionnaire	Online survey
Mansour et al^[28]^	2015	Egypt	To evaluate the informed consent process, misconceptions and motivations among clinical trials participants	Clinical trials participants	103	Cross-sectional survey	Self-administered questionnaire	Clinical research center
Mirzazadeh et al^[29]^	2020	Iran	To understand the perceptions, concerns, barriers and motivators to participation and retention in HIV/HCV cohort studies.	People who inject drugs	30	Qualitative study design	Focus groups	Urban setting
Nabulsi et al^[30]^	2011	Lebanon	To explore the attitudes of parents from a developing country towards child participation in research trials	Parents	33	Qualitative study design	In-depth interviews	Hospital
Nasef et al^[31]^	2014	Egypt	To explore the attitudes and beliefs influencing parents’ decision to involve their children in clinical research	Parents or guardians	357	Cross-sectional survey	Self-administered questionnaire	Hospital
Salem et al^[32]^	2019	Lebanon	To explore the knowledge, attitudes, and perceptions of patients with cancer and their caregivers regarding participation in clinical trials	Patients and caregivers	210	Cross-sectional survey	Interview questionnaire	Outpatient clinics
Tohid et al^[33]^	2017	Qatar	To determine the perceptions toward clinical research among general public	Adults population	2517	Cross-sectional survey	Interview questionnaire	Major public events

### 3.1. Motivators

The included studies identified 6 main motivators of research participation among patients, general public, and patient family members as the most reported (Table 2). The belief that research participation might result in personal benefit was the most cited motivator by almost three quarters 17 (74%) of the studies. This might come in the shape of accessing hospital care or costly drugs that are more effective than the available treatments, drugs of fewer side effects or otherwise unavailable, as well as receiving better treatment or more attention from healthcare providers.^[15,17,21–23,27,28,31,33]^ In addition to that, participation was motivated by having financial gains, expressing their opinions, sharing their complaints, or getting to learn about the scientific topic being researched.^[14,20,21,23,25,28,30,31,33]^ Moreover, few of these studies showed that patients are often motivated to participate in research if they knew in advance that they would have access or receive feedback on their test results.^[12,16,27]^ One study that aimed to assess participation in HIV/HCV cohort studies among people who inject drugs, few participants expressed that among the motivators to be enrolled in the protection from drug-related police interventions during the study period.^[29]^

**Table 2:** Motivators to participation in clinical research.

Motivators	Articles no. (%)	Observed examples
Personal benefits	17 (74)	- Receive better treatment, care, and medical attention, protection.
		- Access to hospital care, new drugs, drugs specific to condition or drugs difficult to obtain, free tests.
		- Monetary incentives/compensation.
		- Share experiences and complaints.
		- Learn about science, research, or health topics.
Help others/altruism	13 (57)	- Improve society health, help other patients, andaltruism.
		- Support scientific advancement, help find cures for diseases with less side effects, and help improve the system.
		- A sense of duty/commitment to community and to volunteering.
		- To advance research of a certain area (e.g., Biomedical research).
Research process	12 (52)	- Study design (i.e., study with no invasive procedures such as questionnaires versus minimally invasive procedures such as blood samples’ collection).
		- Adequate explanation by researchers about disease process, the conducted research and its importance, benefits, hazards, and monitoring plans.
		- Allowing participants time to think before enrollment and obtaining informed consent.
		- Government approval, ethics committee involvement, fairness in the selecting participants, and allowed withdrawal.
		- Good privacy and confidentiality measures.
		- Experienced and local researchers.
Physician influence	8 (35)	- Trust in treating physician’s recommendation or responding to physician’s request to participate.
		- Fear of jeopardizing relationship with physicians and healthcare personnel in case of refusal to participate, and fear of receiving suboptimal medical care.
		- Having the chance to consult family physician and have them look at the study protocol.
		- If initially approached by treating physician.
Family encouragement	5 (22)	- Family encouragement to participate.
		- Presence of family members when approached.
Religious	5 (22)	- Religious permission of samples’ donation and considering research participation a good deed.
		- Presence of a religious representatives in the clinical trial.

In 13 (57%) of the studies, clinical research in the EMRO region was motivated by the desire to help other patients and improve society and its healthcare system. This is driven by a sense of duty and commitment to the country, community, and volunteerism.^[14,20,21,23–28,33]^ Furthermore, the desire to contribute to knowledge and scientific advancement was another driving factor.^[14,15,19,21,24,26–28,30]^

The research process and design were the third common factors influencing the decision of participation in research, which was reported in 12 (52%) of the included studies. Participants were more likely to consent to participate in observational studies in which data are collected through an interview, survey or questionnaire; and in studies that include minimally invasive procedures such as blood sample collection.^[11,16,24]^ Additionally, participation in research was positively influenced by the adequacy of information provided to participants regarding the disease/condition being investigated, the importance of the research and its impact on patient’s health outcomes, the procedures being taken as part of the research, the benefits and potential hazards to expect, and the plan of monitoring the patients during their participation period.^[18,27,31]^ Allowing the participants time to think before enrolling them in research motivated their participation and adequate explanation on their rights to sign an informed consent emphasizing that they will not be subjected to research without their approval and signature of a consent form. In addition to that, they preferred to learn that withdrawal from research is allowed and that the researchers have obtained the necessary governmental approvals.^[11,12,14,15,18,24,27,30,32]^ Informing participants about vital confidentiality and privacy measures throughout the study was an essential component in enrolling in research.^[14,15,27]^

Interestingly, participants in 8 (35%) of the studies reported that they were more likely to enroll in research if this was recommended by their physician, if they had a chance to consult them, or if their physician approached them during the initial recruitment process. Others were motivated by the fear of jeopardizing the relationship with their physicians and healthcare providers or receiving suboptimal medical care if they refused research participation.^[14–18,24,26,28,30]^ Other factors played a role in motivating research participation, such as family encouragement, having a family member around during recruitment, religious permission of research participation, and knowing that God will reward them for this good deed.^[12,14,15,21,25–28]^

### 3.2. Barriers

The main barriers to research participation include fear, the research process, trust, lack of interest or personal benefit, religious concerns, and family or cultural concerns (Table 3). In addition to fear of privacy or confidentiality breach, safety concerns were the most essential and commonly reported barrier in 20 (87%) studies. Participants also shared their fear of receiving suboptimal treatment if they decided to withdraw from research after an initial agreement to participate. Additionally, a misconception that research, especially trials of drugs or vaccines, should be conducted on animals but instead is being done on human beings.^[11,13–16,18–27,30–33]^ Moreover, participants’ fear of discovering they have a disease when they enroll in the study was among the identified barriers.^[29]^

**Table 3:** Barriers to participation in clinical research.

Barriers	Articles No. (%)	Observed examples
Fear/concerns	20 (87)	- Withdrawal from research will result in receiving suboptima medical care.
		- Safety concerns (i.e., fear of adverse effects, fear that new drugs/vaccines that have not been studied on humans are not safe, and misconception that all clinical trials are of new interventions with no established safety on humans).
		- Privacy and confidentiality concerns and fear of information leakage.
		- Fear of the unknown (e.g., discovering they have a disease when they participate in the study).
		- Fear of pain with invasive procedures, and fear of acquiring infections.
Research process	18 (78)	- Study type and design (e.g., less likely to participate in drug clinical trials).
		- lack of awareness of research concepts and concerns regarding the consenting process and patients’ rights in research.
		- Concerns regarding randomization, blinding, multiple visits, recontact by the research team, and the time and effort needed to participate in research.
		- Concerns regarding the associated costs (e.g., transportation) and lack of monetary compensation
Trust	13 (57)	- Mistrust in the healthcare systems and providers (e.g., belief that specimens collected for research purpose without patients’ consent).
		- Concerns regarding medical errors, lack of research supervision, indefinite storage of samples, and sample exploitation.
		- Belief that research is only of interest to clinicians for selfish reasons (e.g., career advancement, monetary rewards)
Lack of benefits/interest	8 (35)	- Research participation has no direct benefit to participants (test results are not shared, no financial compensation).
		- Lack of interest in participation due to stable health or dislike of hospitals and physicians.
Religious concerns	4 (17)	- Religious concerns that research might be tampering with religion or that provided samples might be used for research prohibited by religion.
Family, social, or cultural	2 (9)	- Social and cultural barriers (e.g., visits to medical/research center after working hours or family objects to participation).

The research process played a role and was one of the main barriers in 18 (78%) of the 23 studies. Some study designs were conceived demoralizing by research participants for the same safety concerns, for example, drug clinical trials. Additionally, studies that included randomization and blinding were considered alienating. Time was another main barrier to participation and concerns regarding the logistical issues such as multiple visits and the need for transportation or being re-contacted by the researchers.^[13–15,18,20–33]^

In 13 (57%) studies, views reflecting lack of trust in the healthcare system and providers were observed. Some patients believed that their left-over specimens and samples (after routine surgery, for example) were collected for research purposes without their consent, which demotivated their participation. Others believed that the samples they provide for research might be exploited or stored indefinitely, and hence they prefer not to participate. Furthermore, some believed that research, especially research conducted in EMRO countries, lacks regulatory supervision, and they believed it is only of interest to clinicians as it is crucial for their career advancement.^[12,15–17,19,23,26–28,30–33]^

Additionally, a trend of lack of interest in research participation due to a stable health condition or lack of personal benefit was observed.^[13,15,20,21,26,27,33]^ Last, religious and cultural concerns had a role in discouraging research participation, as observed in a few of the included studies.^[13,15,16,20,21,27]^

## 4. Discussion

In this review, we identified 6 main motivators and barriers to research participation among the population of the EMRO region. This population is unique in its diversity as it includes 22 countries with different ethnic and racial backgrounds and economic statuses. However, this complex population shares enormous similarities due to shared religious and cultural characteristics. Interestingly, some identified factors were interpreted as both motivators and barriers to research participation.

An essential factor that was perceived as a motivator and a barrier is the study design, including the method used for data collection. Participants were most likely to enroll in studies of observational nature, such as questionnaires, and in studies that include minimally invasive procedures.^[11,16,24]^ They were mainly resistant to participating in drug trials, including invasive procedures such as tissue biopsy.^[11,14,23,24,31,32]^

This was opposed by study findings that reported a high proportion of patients considered participation in trials involving repeat biopsies. However, the study included patients with gastrointestinal malignancies or lymphoma only, and their views on biopsies may differ from other patients.^[34]^ Resistance to participate in such trials can be attributed to multiple factors: fear and safety concerns, lack of awareness on concepts of clinical research and its regulations, and mistrust in healthcare providers and in the system. This was confirmed by a global survey about attitudes and experiences of the public toward participation in clinical research. The study included more than twelve thousand individuals, representing 68 countries from North America, South America, Europe, Asia Pacific, and Africa. This study found that the top perceived risk to research participants included the fear of adverse drug events and risks to overall health. Additionally, while >80% of the respondents believed in the importance of research, only 30% were willing to participate.^[35]^

The consenting process is another factor that was perceived as critical by many participants; however, some found the written informed consent complex enough to hinder the participation. Furthermore, we found that some participants believed that they were recruited into research without their knowledge and consent. This was consistent with the findings from a study conducted in Canada. The authors found that about 30% of their study participants were uncertain or believed that clinical research participants are rarely or never informed of their participation.^[36]^

The reimbursement for research participation was another area of controversy. It was encouraging to some, offensive or unfamiliar to others, and some found it scary. “So, all parents will be scared, especially because the vaccine is provided for free when we know it is expensive! When we come here, they give us money for our transportation! This is scary, you know!!” a mother stated regarding her children’s participation in clinical research.^[30]^

In contrast, a qualitative study by Breitkopf et al involved thirty women from Texas, United States of America, who were interviewed following their participation in a clinical trial to explore their perceptions regarding reimbursement in clinical research. The participants perceived it as a positive addition that would encourage participation. They also believed that the time spent, the inconvenience and sensitivity of the research topic and the possible hazards reimbursement amount should be reflected on the monetary amount being reimbursed. It was not perceived that offering monetary compensation is likely to coerce participation in research if the individual believed the research to be unacceptable or of high associated risks.^[37]^

Religion was a motivator for participation in research for those who viewed their participation as essential to advance science and help others and for those who perceived it as a means of pleasing God through a good deed act. On the other hand, some were doubtful that their samples might be used in research that is not permitted by religion. Another study conducted in the United States assessing the attitudes of Muslim immigrant women toward cervical cancer screening showed that many participating women expressed their resistance to practices that challenge their religious and cultural values.^[38]^

Our review also showed that effective communication with potential participants was an important factor in encouraging their enrollment in clinical research. This includes providing adequate information about the importance of research and its impact on patient’s health outcomes, research procedures, benefits and potential hazards, and the participants’ rights throughout the process. This is likewise shown in a study conducted in California, United States of America, which evaluated awareness level and willingness to participate in cancer clinical trials among 1188 patients and their families. They reported a significant positive correlation between awareness and willingness to participate in the trials.^[39]^

Despite that, effective communication was not always practiced to its best. There was an inconsistent across studies regarding the provided information to participants. In a study conducted in the United Kingdom and included 486 cancer patients, most of the participants (95%) reported that they were provided with enough information about the trial.^[40]^ In Burns et al study, it was reported that only about 70% of participants were adequately informed about the risks and benefits of study.^[36]^

Evaluating the factors influencing the perception and attitude towards participation in clinical research is crucial for the research in the region. Nevertheless, this could be challenging to be assessed precisely. In our review, there are several important strengths and limitations. This review comprehensively addressed the factors affecting participation in clinical research from studies conducted in the region. Among the limitations is the quality of data due to the observational cross-sectional design of the included studies.

Additionally, all the studies depended on self-reporting methods, which might bias the results depending on the participant’s ability to recall or the desire to share what they believe they prefer to hear with the researchers. The majority of the included studies were based on approaching the participants giving a hypothetical scenario asking about their perceptions of participation in clinical research if they were contacted to be enrolled. This creates a gap between the hypothesis and the real world. However, these studies elucidate the factors that motivate or discourage participation in clinical research.

## 5. Conclusion

The included studies identified the motivators and barriers to clinical research participation among the patients and the general public in the EMRO countries. The identified factors are essential to consider when planning for future research. As they explain what motivates participation and explore some of the fears of the target population, these factors are important to address during the planning stage. There might be a gap between theory and practice due to the hypothetical design of most studies included in this review. Hence, future research to assess the perceptions and attitudes of patients who are actually enrolled in clinical research or those who are rejected to participate is further needed. Moreover, it is important to investigate the association of these factors and the likelihood of individuals participation in clinical research.

## Author contributions

All authors of this review have participated to this article with different roles. M.A. provided the idea for the article and prepared the review proposal. B.I. provided supervision and guidance for the review concept, proposal, planning, and execution. I.A. and M.A. performed the literature search, data analysis, and initial draft. T.S. validated the review findings through a final assessment discussion with M.A. regarding the search strategy, selection criteria, data extraction and analysis, and quality assessment. M.I.B., I.A., and M.A. prepared the final manuscript version through critical review.
